# Comparison of three multichannel TX/RX coils for anatomic and functional CMR at 7.0T

**DOI:** 10.1186/1532-429X-15-S1-W24

**Published:** 2013-01-30

**Authors:** L Winter, P Kellman, W Renz, A Graessl, F Hezel, C Thalhammer, F von Knobelsdorff-Brenkenhoff, V Tkachenko, J Schulz-Menger, T Niendorf

**Affiliations:** 1 Berlin Ultrahigh Field Faciltiy (BUFF), Max Delbrück Center for Molecular Medicine, Berlin, Germany; 2 Department of Cardiology and Nephrology, HELIOS Klinikum Berlin-Buch, Berlin, Germany; 3 Experimental and Clinical Research Center, A joint cooperation between the Charité Medical Faculty, Berlin, Germany; 4 Laboratory of Cardiac Energetics, National Institutes of Health/NHLBI, Bethesda, MD, USA; 5 Siemens Healthcare, Erlangen, Germany

## Background

Ultrahigh field cardiac MR (CMR) is an area of vigorous ongoing research and is regarded as one of the most challenging MRI applications since image quality is not always exclusively defined by signal-to-noise ratio (SNR) and contrast-to-noise ratio (CNR). The progress in the field has been driven by pioneering explorations into hardware developments which focused on novel multichannel transmit and receive coil arrays technology to tackle the challenges of B1-field inhomogeneities. Consequently, transmit-receive (TX/RX) structures are not a nicety but a necessity for ultrahigh field CMR including a trend towards a large number of transmit and receive channels. For all these reasons, this study was designed to compare the quality of anatomical and functional CMR at 7.0 T under clinical aspects using cardiac optimized transceive coils that use loop structures with the number of TX/RX elements ranging from 4-16.

## Methods

Ten volunteers [seven males; BMI 21.4±2.8 kg/m2] underwent CMR. 4-ch, 8-ch and 16-ch TX/RX coils that use loop elements were implemented. All volunteers were examined with all three coils. For each subject, two-, three-, and four-chamber standard views of the left ventricle and a set of short-axis views ranging from the atrioventricular ring to the apex were acquired using a 2D CINEFLASH [FOV360×326mm2, TE 2.7 ms, TR 5.6 ms, 444 Hz/px, 30 cardiac phases, 8 views per segment, slice gap 2 mm, 1.4×1.4×4mm3. End-diastolic and endsystolic volume, LV ejection fraction, and left ventricular mass were obtained. To evaluate parallel imaging performance, geometry factor maps were calculated for reduction factors (R=1-4). For SNR/CNR assessment the 2D CINE FLASH technique included a preliminary noise sequence to measure the noise-correlation matrix and the image data was reconstructed in SNR units. Overall image quality was scored in a blinded consensus reading of two experienced CMR readers based on blood/myocardium contrast, anatomic border sharpness, and visualization of subtle anatomical features.

## Results

Mean total examination time was 29±5 min. All images obtained with the 8- and 16-channel coils were diagnostic. No significant difference in ejection fraction (EF) (P>0.09) or left ventricular mass (LVM) (P>0.31) was observed between the coils. The 8- and 16-channel arrays yielded a higher mean SNR in the septum versus the 4-channel coil. The lowest geometry factors were found for the 16-channel coil (mean ± SD 2.3±0.5 for R=4). Image quality was rated significantly higher (P<0.04) for the 16-channel coil versus the 8- and 4-channel coils.

## Conclusions

All three coil configurations are suitable for CMR at 7.0T under routine circumstances. A larger number of coil elements enhances image quality and parallel imaging performance but does not impact the accuracy of cardiac chamber quantification.

**Figure 1:**
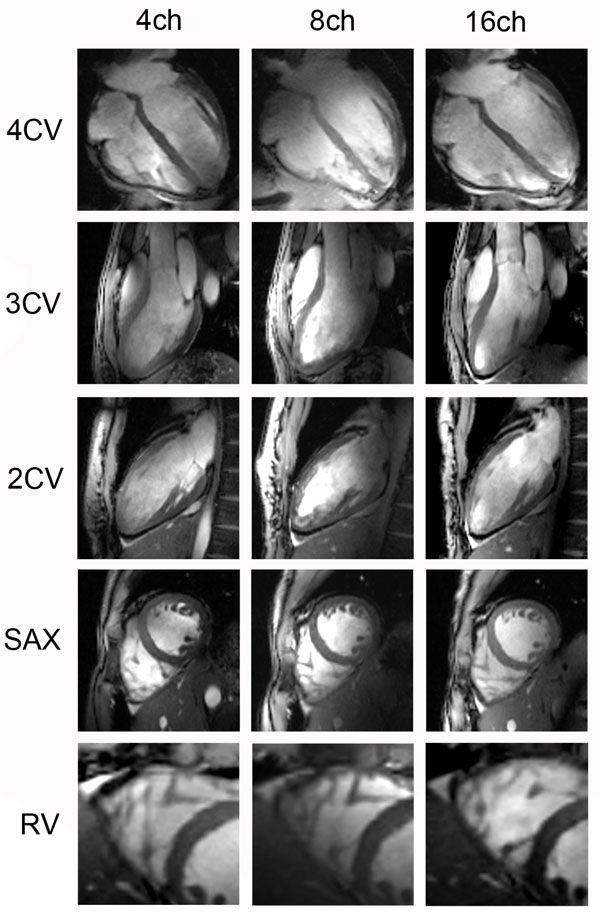
2D CINE FLASH images for the 4-channel (left), 8-channel (middle), and 16-channel (right) TX/RX coil arrays derived from the same subject. A four-chamber view (4CV), three-chamber view (3CV), two-chamber view (2CV), short-axis view (SAX), and a magnified view of subtle anatomic details of the right ventricle (RV) at end-diastole are displayed. The images were not corrected for receive inhomogeneity.

**Figure 2:**
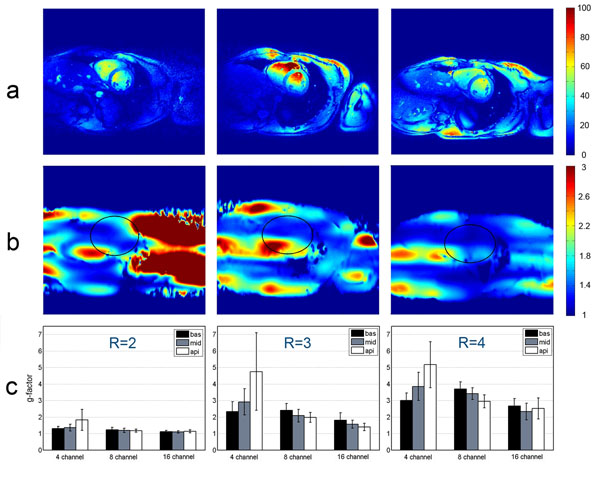
**Survey of the parallel imaging performance of the 4-channel, 8-channel, and 16-channel TX/RX coil.** a) Quantitative SNR maps observed for GRAPPA reconstruction using an acceleration factor of R=4 for the 4-ch (left), the 8-ch (middle), and the 16-ch TX/RX (right) coil. b) Geometry factor maps derived from GRAPPA reconstruction for an acceleration factor of R=4 for the 4-channel (left), the 8-channel (middle), and the 16-channel (right) TX/RX coil with a region-of interest indicating the position of the heart. c) Comparison of gfactor (mean value and standard deviation) obtained for the 4-, 8-, and 16-channel TX/RX coils for left basal, middle midventricular, and right apical short-axis views

